# Neurofilament light and glial fibrillary acidic protein do not reflect neuronal or glial damage during different intracranial radiotherapy regimes: a pilot study

**DOI:** 10.3389/fonc.2026.1745704

**Published:** 2026-06-08

**Authors:** Yvonne Dzierma, Holger Sebb, Michael Utzig, Nurlan Abdullayev, Christian Berdel, Christian Ruebe, Jochen Fleckenstein, Markus Hecht, Guido Hildebrandt, Mathias Jucker, Kristina Heyne

**Affiliations:** 1 Klinik und Poliklinik für Strahlentherapie, Universitatsmedizin Rostock, Rostock, Germany; 2 Klinik für Strahlentherapie und Radioonkologie, Universitätsklinikum des Saarlandes, Homburg, Germany; 3 Klinik für Strahlentherapie und Radioonkologie, Universitätsklinikum Hamburg-Eppendorf, Hamburg, Germany; 4 Klinik für Strahlentherapie, Westpfalz-Klinikum, Kaiserslautern, Germany; 5 Hertie-Institute for Clinical Brain Research, University of Tübingen, Tübingen, Germany; 6 Biobank der Medizinischen Fakultät, Universität des Saarlandes, Homburg, Germany

**Keywords:** glial fibrillary acidic protein (GFAP), neurofilament light (NfL), neurooncology, radiotherapy, stereotactic radiosurgery

## Abstract

**Introduction:**

Neurofilament light chain (NfL) and glial fibrillary acidic protein (GFAP) have been shown to be non-specific markers of central nervous system damage and have been associated with the occurrence of primary or secondary brain tumours. However, the response of these markers to cerebral radiotherapy has not been explored to date.

**Methods:**

In this pilot study, we measured NfL and GFAP levels before, during, and after radiotherapy, as well as during follow-up, in a representative clinical cohort of patients treated with percutaneous radiotherapy either for single or multiple brain metastases, for glioblastoma, or with whole-brain irradiation.

**Results:**

We confirmed elevated NfL and GFAP levels in all patients before the start of radiotherapy. A significant increase after the onset of radiotherapy was not observed. During long-term follow-up, most cases of decreasing NfL and GFAP values correlated with treatment response, whereas the most pronounced increases in serum NfL levels were followed by the detection of several new cerebral lesions.

**Conclusion:**

Cerebral radiation injury does not appear to correlate with increased NfL and GFAP levels during radiotherapy. After treatment, NfL and GFAP remain suitable candidates for follow-up and possibly for the early detection of new lesions.

## 1 Introduction

Approximately 20–40% of patients with solid cancers develop cerebral metastases during the course of their disease, with rising incidence owing to improved therapeutic options and prolonged survival (1). For patients with limited intracranial disease, stereotactic radiotherapy (SRT) is the mainstay of treatment, either as single-session radiosurgery (SRS) or fractionated stereotactic radiotherapy (fSRT). SRT has been shown to achieve local control at 12 months of more than 90% (e.g., 2, 3), with generally good tolerability. For multiple brain metastases, whole-brain radiotherapy (WBRT) was previously the standard of care (1) and has since been refined with sophisticated techniques such as hippocampal sparing or simultaneous integrated boost (SIB) to larger lesions (4). However, recent technological advances allow stereotactic treatment of multiple brain metastases in a single session (5–11), and growing evidence suggests that the repeated use of SRT in combination with frequent magnetic resonance imaging (MRI) follow-up may be equally effective in terms of intracranial control and overall survival, while being associated with better preservation of neurocognitive function and, consequently, improved quality of life (12–21).

The second remaining indication for WBRT is prophylactic cranial irradiation (PCI) in patients with limited-disease small-cell lung cancer (SCLC) to prevent cerebral dissemination (22). In this context, a strategy of close surveillance and stereotactic treatment of newly arising lesions appears similarly effective in terms of overall survival (23, 24), provided that frequent MRI is performed.

The availability of MRI is, however, very limited in routine clinical practice for many patients. In addition to the difficulty of obtaining MRI time slots in many regions, even in highly developed nations, an MRI examination poses organisational challenges, is physically demanding for terminally ill patients, and may be associated with psychological distress. It would therefore be desirable to establish a serum marker sufficiently sensitive to identify those patients who require urgent MRI, in order to prioritise examinations and appointments.

Neurofilament light chain (NfL) is a structural protein of mature myelinated axons that is released into the cerebrospinal fluid and, at lower concentrations, into the blood in cases of neuronal damage (25). Since the introduction of highly sensitive assays for measuring NfL in blood samples, this marker has been evaluated in a wide range of neurological pathologies (e.g., see reviews 26–28).

In cases of traumatic brain injury, a temporal pattern is observed, with increasing NfL concentrations over a period of approximately 1–2 weeks, followed by a return to normal levels over 12–15 months. Furthermore, the rise in NfL correlates with the severity of the trauma as well as with poorer outcomes (29–31). A similar time course has been observed in acute ischaemic stroke, in which higher NfL levels were likewise associated with persistent cognitive and motor impairments (32, 33). Comparison with MRI data also revealed a correlation between NfL levels and ischaemia volume, as measured on the T2 fluid-attenuated inversion recovery sequence (34). In patients with multiple sclerosis, higher NfL levels are associated with increased brain atrophy over time (35), and serum levels during disease-modifying therapy are being investigated in early studies as markers of treatment response (36). In patients with Alzheimer’s disease, NfL has demonstrated predictive value for cognitive decline and hippocampal atrophy even at the preclinical stage (37, 38). Similar findings have been reported in patients with Parkinson’s disease and other neurodegenerative conditions (39), as well as in other cerebral injury processes such as alcohol dependence (40). Overall, NfL appears to be a sensitive, albeit non-specific, marker of neuronal damage.

While initial studies have reported that NfL appears to be increased in patients with primary or secondary tumours of the central nervous system (41–44), to our knowledge, the behaviour of this marker during radiotherapy has not yet been thoroughly investigated.

At the molecular level, it would be reasonable to assume that NfL and glial fibrillary acidic protein (GFAP) might increase during radiotherapy, as damage to neurons or glial cells may result in the release of these intracellular molecules, which should be measurable in peripheral blood. Any disruption of the blood–brain barrier—caused either by the tumour itself or potentially by radiotherapy—would further increase peripherally measured serum levels. In particular, we hypothesised that ablative radiation doses, such as those applied in stereotactic radiosurgery, might cause greater cellular damage and, consequently, increased NfL and GFAP release, possibly more so than fractionated radiotherapy regimens, such as 30 fractions of 2 Gy administered in glioblastoma treatment, and even more so than lower total-dose, fractionated whole-brain radiotherapy. The biological rationale for this assumption is that normofractionated radiotherapy primarily aims at clonogenic cell inactivation, rather than ablative treatment, without necessarily causing immediate cell death leading to the release of intracellular molecules.

To investigate the influence of different radiotherapy treatment regimens on NfL and GFAP serum levels, we performed a pilot study in a clinically representative cohort of patients with cerebral malignancies undergoing radiotherapy. We included patients with a single or multiple brain metastases treated with stereotactic radiotherapy either as a single-modality treatment or postoperatively; patients receiving whole-brain radiotherapy either for multiple brain metastases (therapeutic WBRT) or as PCI; and patients with glioblastoma (GBM) or similar tumours receiving concomitant radiotherapy according to the Stupp or CeTeG protocols (45, 46). In addition to NfL as a marker of axonal damage, we analysed GFAP as a marker of glial damage (e.g., 47 and references therein).

The main questions posed in this initial exploratory analysis are:

1. Are baseline levels of these markers elevated before the start of radiotherapy?
2. How do the levels change over the course of radiotherapy? Can an increase in serum levels be observed during or after radiotherapy, and if so, does this differ between fractionation regimens (e.g., WBRT vs SRT)?
3. What is the behaviour of these values during longer follow-up? Do they return to normal levels? Can they be used as early markers of relapse or disease progression?

Although this study provides only a preliminary assessment in a limited number of patients, we believe that addressing these questions may provide important input for future treatment strategies in radiotherapy and oncology for primary and secondary brain tumours, or at least pave the way for further focused studies in this area.

## 2 Patients and methods

We included 48 patients treated with cerebral radiotherapy at the Department of Radiotherapy and Radiation Oncology, Saarland University Medical Center, between May 2023 and May 2024. All patients provided written informed consent for blood sampling and subsequent analysis. The diagnostic and treatment workflow were not influenced by participation in this observational trial. The trial was conducted in accordance with the Declaration of Helsinki and was approved by the local ethics committee (Ärztekammer des Saarlandes) under reference number 190/22.

Several patients were lost to follow-up after the first treatment session. Their data were included in the analysis of pre-radiotherapeutic NfL and GFAP levels, as well as potential increases on day 1; however, longer follow-up data were not available. The final patient cohort included in the complete analysis comprised 26 patients:

• 11 patients with brain metastases, of whom 4 had undergone previous surgery of at least one metastasis (2 patients received a second session of SRT without prior resection), and 7 received SRT for 1–28 brain metastases without prior surgery (1 patient underwent repeated SRT courses).
• 7 patients treated with WBRT, of whom 3 received PCI (2 with hippocampal sparing (HCS)) and 4 received therapeutic WBRT (1 with SIB). Of these, follow-up longer than 30 days after completion of radiotherapy was available for only 3 patients (2 with WBRT or WBRT + SIB and 1 with PCI + HCS). Inclusion of these patients in the trial was limited because the institutional protocol changed from WBRT and PCI to SRT for multiple metastases between submission of the ethics proposal and study initiation, resulting in only very few patients receiving WBRT thereafter.
• 8 patients treated according to a protocol for GBM (6 histologically confirmed GBM, 1 diffuse glioneuronal tumour with oligodendroglioma-like features (DGONC), and 1 gliosarcoma). Of the patients with GBM, three had only short follow-up (less than 30 days after the last radiotherapy session).

The demographic and clinical characteristics of the participants are presented in **Table 1**.

**Table 1:** Demographic and clinical characteristics of the patients included in this study.

Study group	SRT without surgery (n = 8)	SRT post-surgery (n = 5)	WBRT therapeutic (n = 4)	PCI (n = 3)	GBM (n = 8)
Age (mean, range)	64.5 (58–69)	67.2 (64–70)	60.0 (57–67)	65.7 (61–70)	61.4 (51–74)
Gender (F/M)	4/4	2/3	2/2	2/1	2/6
Diagnoses	NSCLC (4) Melanoma (2) Breast cancer (1)	NSCLC (3) Oesophageal cancer (1) Melanoma (1)	Melanoma (1) SCLC (2) NSCLC (1)	SCLC (3)	GBM (6) Gliosarcoma (1) DGONC (1)
Total (n = 28):	Age 63.6 (51–74) Gender (F/M) 12/16				

SRT, stereotactic radiotherapy; WBRT, whole-brain radiotherapy; SCLC, small-cell lung cancer; NSCLC, non-small-cell lung cancer; GBM, glioblastoma; DGONC, glioneuronal tumour with oligodendroglioma-like features.

Radiotherapy was delivered according to our institutional standard protocol following presentation of each patient case at the interdisciplinary tumour board of Saarland University. All stereotactic treatments were performed using a Varian TrueBeam linear accelerator equipped with a Millennium multi-leaf collimator, delivering 6 MV photons in either flattened or flattening-filter-free (FFF) mode, with patient immobilisation achieved using a thermoplastic mask designed for stereotactic treatment. Patient positioning was verified using the Brainlab ExacTrac system, incorporating surface and thermal cameras as well as orthogonal imaging for each treatment arc. For the simultaneous treatment of multiple brain metastases with a single isocentre, the Brainlab Elements MultipleBrainMets software was used, as previously described (48). Treatment plans for single brain metastases, WBRT, and GBM were generated using the Varian Eclipse treatment planning system. For WBRT and GBM, patients were treated on one of two energy-matched TrueBeam linear accelerators or on one of two matched Varian Halcyon systems.

Following inclusion in the trial, blood samples were obtained before the first radiotherapy session (ideally on the same day, prior to irradiation) and again on the following day. Subsequent laboratory assessments were intended for day 5, day 10, and so forth; however, these were limited to days on which patients attended the clinic (for follow-up, MRI, immunotherapy, etc.), as no funding was available to cover travel expenses for study-related visits. Consequently, complete longitudinal blood sampling was not feasible for all patients.

Patients’ blood samples were collected in S-Monovette 2.7 ml K3E tubes (Sarstedt, #05.1167) and centrifuged at 1,600 × g using an Eppendorf 5810 R centrifuge at room temperature for 10 minutes. The supernatant (plasma fraction) was removed and stored at −80°C. The samples were shipped frozen on dry ice to the analysis laboratory in Tübingen. For NfL and GFAP measurement, serum samples were thawed on wet ice for one hour. Subsequently, they were mixed for 30 seconds and centrifuged for 5 minutes at 10,000 × g and 4°C. Measurements were performed using a single molecule array platform (Simoa HD-X analyser; Quanterix) with commercially available assay kits (Simoa Neurology 2-Plex B Kit (NF-Light/GFAP), Cat. 103520). All samples were measured in duplicate. Plasma samples were automatically diluted 1:4 with Simoa NfL/GFAP sample diluent. Inter-assay variability was evaluated using three specific human samples. All samples were measured in a blinded manner. All but three samples had a coefficient of variation < 20% (maximum 68.8%), and two samples had only one technical replicate. However, remeasurement was not performed for these five samples due to insufficient remaining volume for repeat analysis. The average of the two technical replicates was used for further analysis.

NfL and GFAP levels for each participant are presented in **Table 2**, together with the fold increase relative to the 95th percentile of NfL as published in (49). To our knowledge, no established reference values for GFAP have been published to date.

**Table 2:** NfL and GFAP levels by treatment group.

Group	SRT w/o OP	SRT + OP	WBRT	WBRT w/o OP	PCI	GBM
NfL [pg/mL /-fold increase over 95% percentile]	53.85 / 3.59	49.51 / 2.48	242.34 / 12.12	24.15 / 1.61	42.99 / 2.15	88.99 / 5.93
	24.38 / 1.22	122.95 / 6.15	24.15 / 1.61	247.69/ 16.51	53.24 / 2.66	13.67 / 0.91
	26.02 / 1.30	121.44 / 3.47	247.69 / 16.51	17.23 / 1.15	25.9 / 0.74	70.03 / 3.50
	16.78 / 0.84	175.55 / 5.02	17.23 / 1.15	99.87 / 4.99		58.67 / 3.91
	42.39 / 2.83	256.97 / 12.85	99.87 / 4.99			273.93 / 7.83
	290.41 / 14.52					108.46 / 5.42
	13.68 / 0.68					63.72 / 1.82
	80.39 / 4.02					48.26 / 3.22
Median	34.21 / 2.06	122.95 / 5.02	99.97 / 4.99	62.01 / 3.30	42.99 / 2.15	66.88 / 3,71
GFAP (pg/mL)	193.87	72.08	390.64	280.92	264.33	1441.78
	83.85	339.76	280.92	3090.74	317.99	192.69
	182.34	284.19	3090.74	62.22	83.08	172.99
	64.35	336.09	62.22	1143.54		299.29
	126.21	402.44	1143.54			946.22
	176.48					254.81
	97.46					411.65
	454.89					123.57
Median	151.35	336.09	390.64	712.23	264.33	277.05

All statistical analyses were carried out using Origin 2024. For statistical testing, the non-parametric Wilcoxon test was applied to single or paired data, and the Mann–Whitney test was applied to unpaired data. A p value of 0.05 was considered to indicate statistical significance.

## 3 Results

### 3.1 NfL and GFAP baseline values before radiotherapy

**Figure 1:**
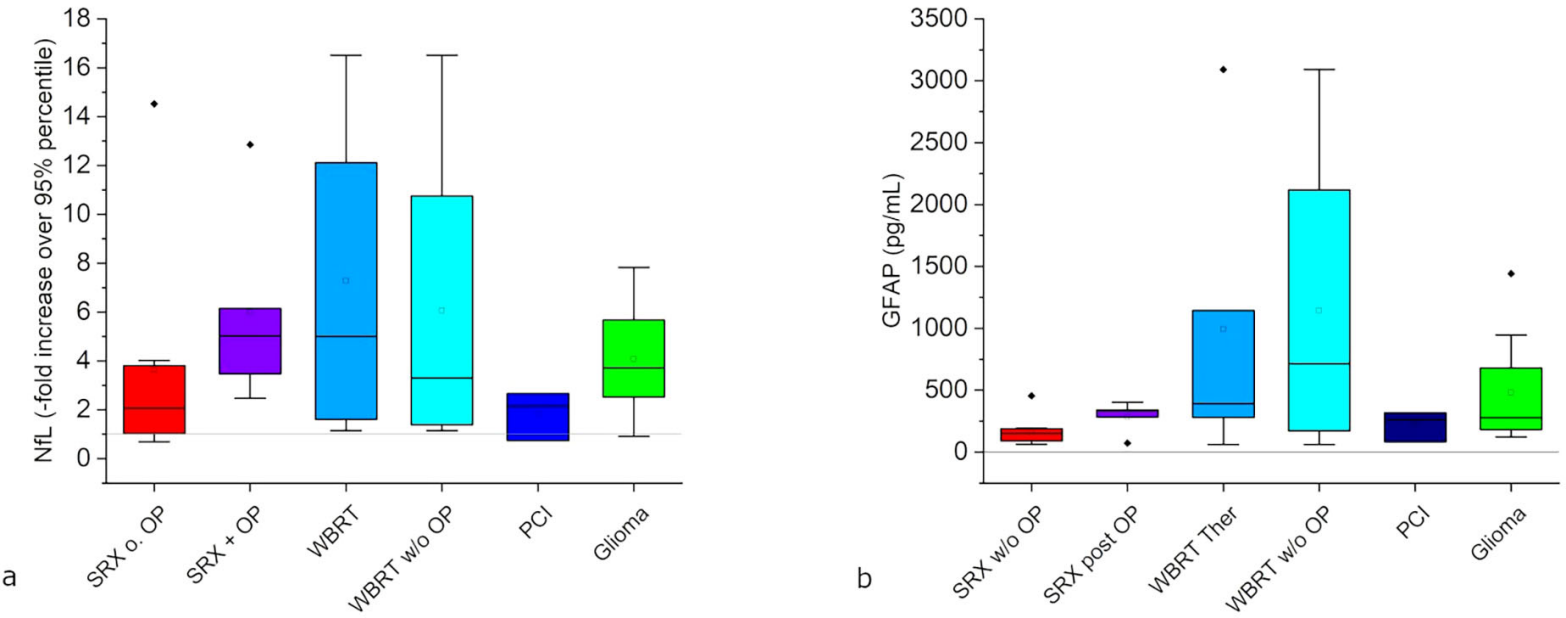
**(A)** Distribution of NfL concentrations before the start of radiotherapy expressed as fold increase over the 95th percentile of reference values. **(B)** Distribution of GFAP serum levels (pg/ml) before the start of radiotherapy.

For all patients in this study, NfL values were elevated above the median reference value (50th percentile) before the start of radiotherapy (see **Figure 1A**). When analysing the different patient cohorts separately, NfL values were significantly elevated above the 95th percentile in all groups (SRT without surgery: p = 0.041; SRT after surgery: p = 0.0038; therapeutic WBRT: p = 0.0038; excluding the patient with prior surgery, therapeutic WBRT: p = 0.011; GBM: p = 0.0042), except for the PCI group (p = 0.32, not significant).

In general, NfL appeared to be more elevated in patients receiving post-operative SRT than in those without prior resection; however, the comparison between these two groups did not reach statistical significance. One patient treated with therapeutic WBRT had previously undergone resection of one metastasis (NfL-20). As neurosurgery was assumed to be associated with an increase in NfL, similarly to cerebral trauma, **Figure 1** also presents NfL values after exclusion of this patient (WBRT without surgery). This exclusion did not result in a relevant change in the data, as the remaining group of patients still exhibited a significant increase in NfL above the 95th percentile (p = 0.011). Patients treated with therapeutic WBRT, irrespective of whether the post-surgical patient was excluded, presented with higher NfL serum levels before therapy than those receiving PCI, possibly reflecting metastatic burden; however, this difference did not reach statistical significance.

Notably, four patients had baseline values within ±20% of the 95th percentile, while still considerably above the median reference level: NfL-11, NfL-29, NfL-22, and NfL-39. These cases corresponded to the patient with DGONC (who had also undergone surgical resection of the tumour approximately 7 weeks before initiation of radiotherapy), one patient receiving PCI (with no known intracerebral metastatic burden), one patient with four metastases treated with SRT without prior surgery, and one patient with multiple metastases.

When considering GFAP values before commencement of radiotherapy (**Figure 1B**), the distribution pattern across the separate treatment groups appeared to resemble that of NfL. As no established reference values exist for GFAP, absolute concentrations are presented rather than fold changes.

**Figure 2:**
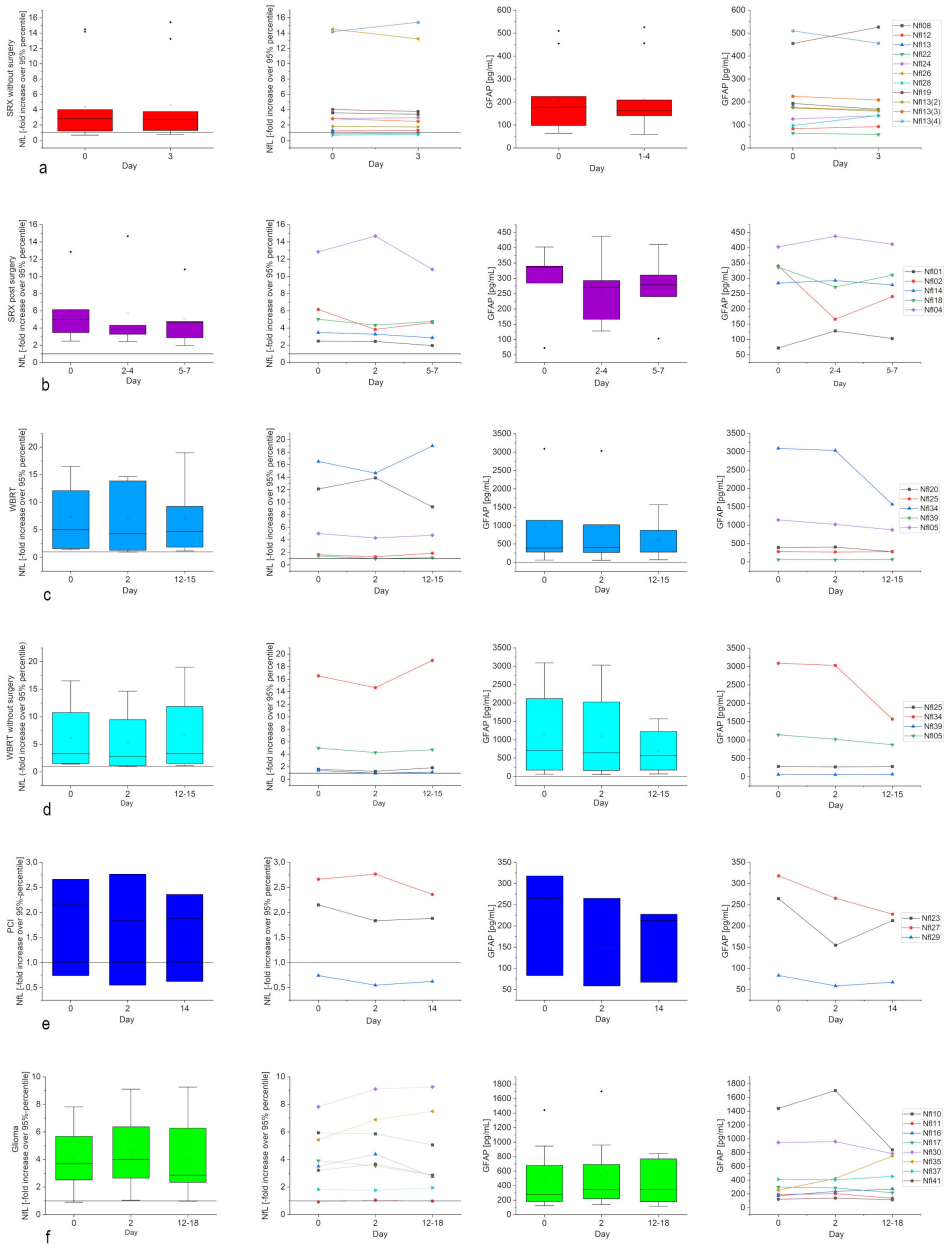
**(A)** NfL and GFAP levels before and after initiation of treatment in patients undergoing stereotactic radiotherapy without prior surgery, including one patient who received three additional treatment courses. **(B)** NfL and GFAP levels before and after initiation of treatment in patients undergoing stereotactic radiotherapy following neurosurgical tumour resection. **(C)** NfL and GFAP levels before and after initiation of treatment in patients receiving therapeutic whole-brain irradiation. **(D)** NfL and GFAP levels before and after initiation of treatment in the same patient group as in **(C)** after exclusion of one case with prior surgery. **(E)** NfL and GFAP levels before and after initiation of treatment in patients with SCLC receiving prophylactic whole-brain irradiation. **(F)** NfL and GFAP levels before and after initiation of treatment in patients with glioblastoma. All serum levels have an uncertainty margin of ± 20%. However, for clarity of visualisation, error bars were not included in the graphical representations.

To evaluate changes in NfL and GFAP following the onset of radiotherapy, samples obtained after one treatment fraction were compared with those collected before initiation of treatment (“day 0”) (**Figures 2A–F**). For some patients, the first post-treatment sample could only be obtained as late as day 4 because a weekend intervened. No significant rise in NfL or GFAP was demonstrated in any of the patient cohorts.

Visual inspection of the data (**Figure 2B**) suggested that, in patients receiving post-operative SRT, NfL values decreased from day 0 to the first measurement following initiation of radiotherapy. This change did not reach statistical significance, as this cohort comprised only five patients, which represents an insufficient sample size to achieve significance in the non-parametric Wilcoxon test. However, this pattern appears biologically plausible, since the NfL increase associated with surgery would be expected to decline over time after the intervention.

Similarly, to account for a possible delayed increase in NfL or GFAP, samples obtained around day 10 were compared with those collected before the start of radiotherapy. Again, no significant increase in NfL or GFAP was observed. As patients undergoing single-session radiosurgery were discharged after the weekend, the next blood sample could only be obtained at the subsequent follow-up visit 8–12 weeks after therapy; therefore, no short-term data were available for this cohort.

In summary, the available data do not indicate an increase in either NfL or GFAP following the start of radiotherapy.

### 3.2 Individual patient cases and follow-up

In this section, selected patient cases are discussed individually. This exploratory analysis is presented for completeness and to illustrate the temporal course of NfL and GFAP, without implying statistical significance.

**Figure 3:**
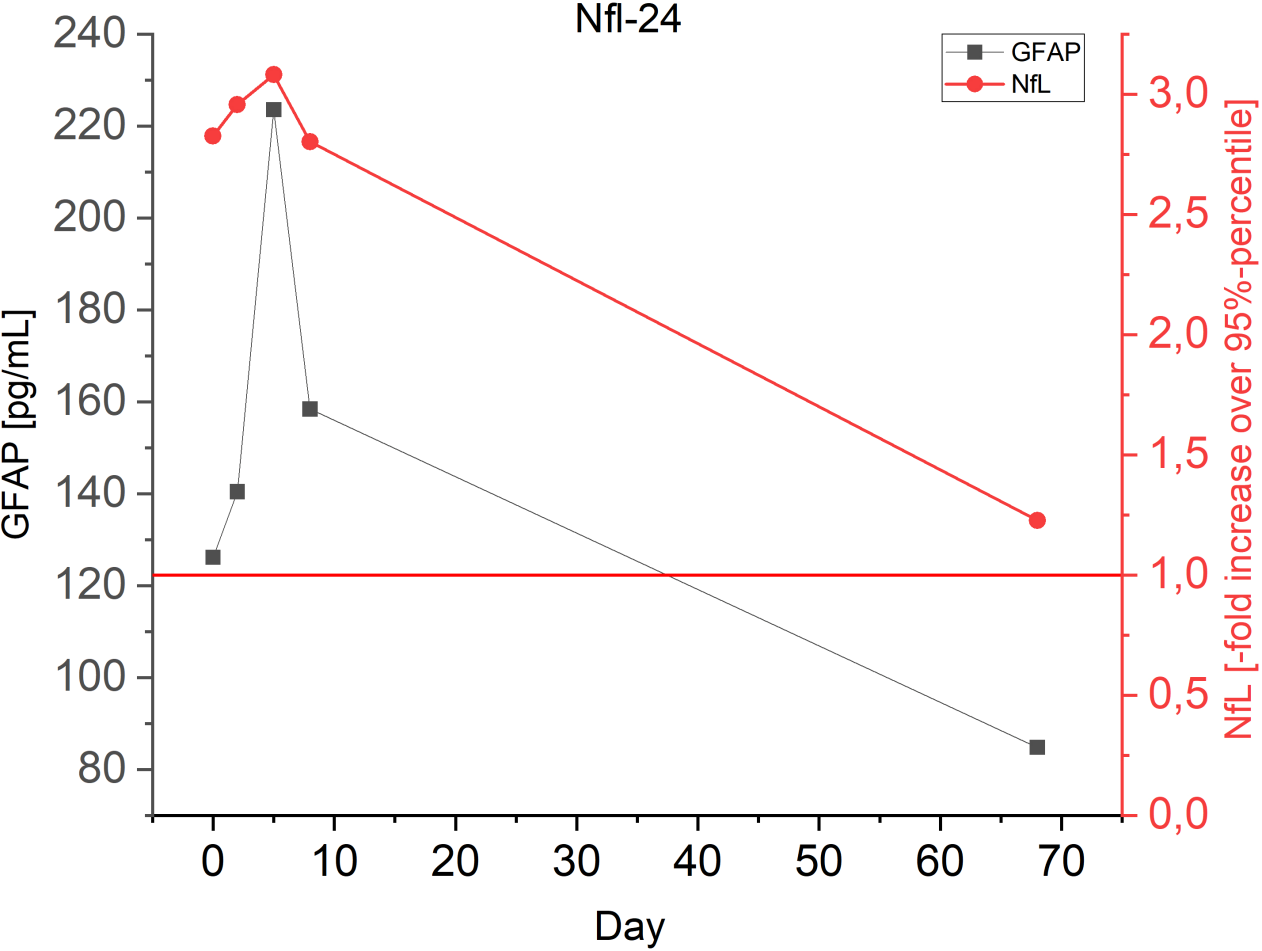
Patient NfL-24 (SRT without prior surgery).

First, patients treated with SRT for cerebral metastases without prior surgery are considered. NfL-24 (**Figure 3**) was a 58-year-old patient with two asymptomatic cerebral metastases from non-small-cell lung cancer (NSCLC), treated in a single radiosurgery session (single-isocentre dynamic conformal arc therapy, 6 MV FFF photons, 25 Gy point dose, with the 80% isodose encompassing 20 Gy). NfL was elevated above the 95th percentile before initiation of therapy, increased slightly (1.09-fold) by day 5, and decreased to just above the 95th percentile at follow-up on day 68. Follow-up cerebral MRI (cMRI) demonstrated good treatment response with no new lesions; the two treated lesions had regressed but remained residually visible. GFAP increased from 126.21 pg/mL before initiation of therapy to a peak of 223.57 pg/mL on day 5, representing a 1.77-fold increase, then decreased to 158.43 pg/mL by day 8, followed by a more gradual decline by day 68. Despite the differing absolute scales, the temporal pattern of GFAP closely mirrored that of NfL.

**Figure 4:**
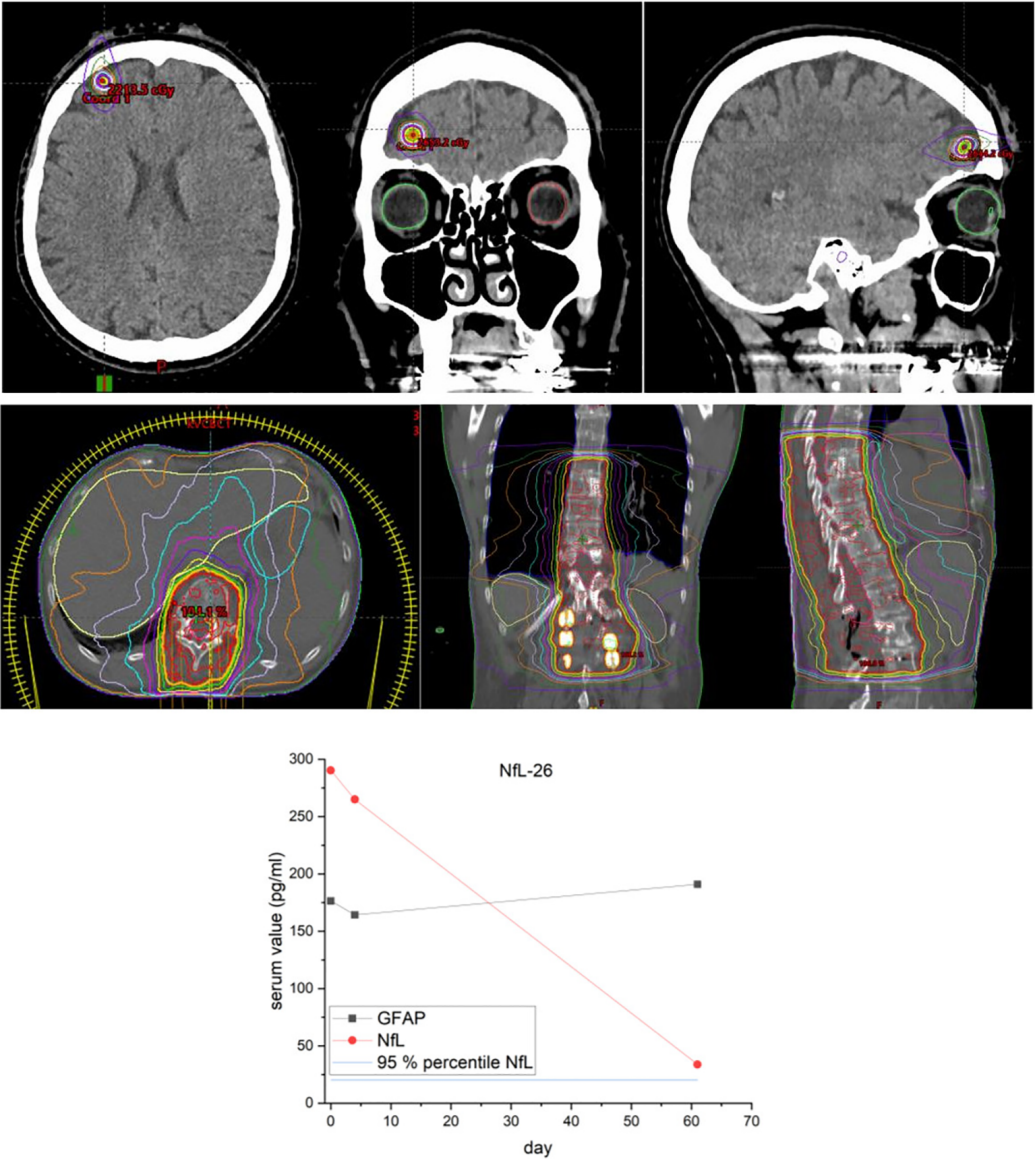
Patient NfL-26: NfL and GFAP values together with dose distributions for spinal irradiation (Th8–L3 for osseous metastases, 30 Gy in 10 fractions of 3 Gy) and dose distribution for cranial radiosurgery (1 × 20 Gy to the surrounding 80% isodose for a single brain metastasis without prior resection).

A similar pattern was observed for NfL-26 (**Figure 4**), a 69-year-old patient with metastatic breast cancer and one asymptomatic cerebral metastasis. Again, NfL decreased to nearly the 95th percentile by day 61 after treatment; however, the baseline NfL value before initiation of radiotherapy was considerably higher in this patient (290.4 pg/mL) than in NfL-24 (42.4 pg/mL). A possible explanation is that this patient also received spinal irradiation (10 × 3 Gy to Th8–L3), which had commenced before the first blood draw (**Figure 4** shows both dose distributions). For this patient, follow-up cMRI also demonstrated good response with no new metastases, consistent with the decrease in NfL.

**Figure 5:**
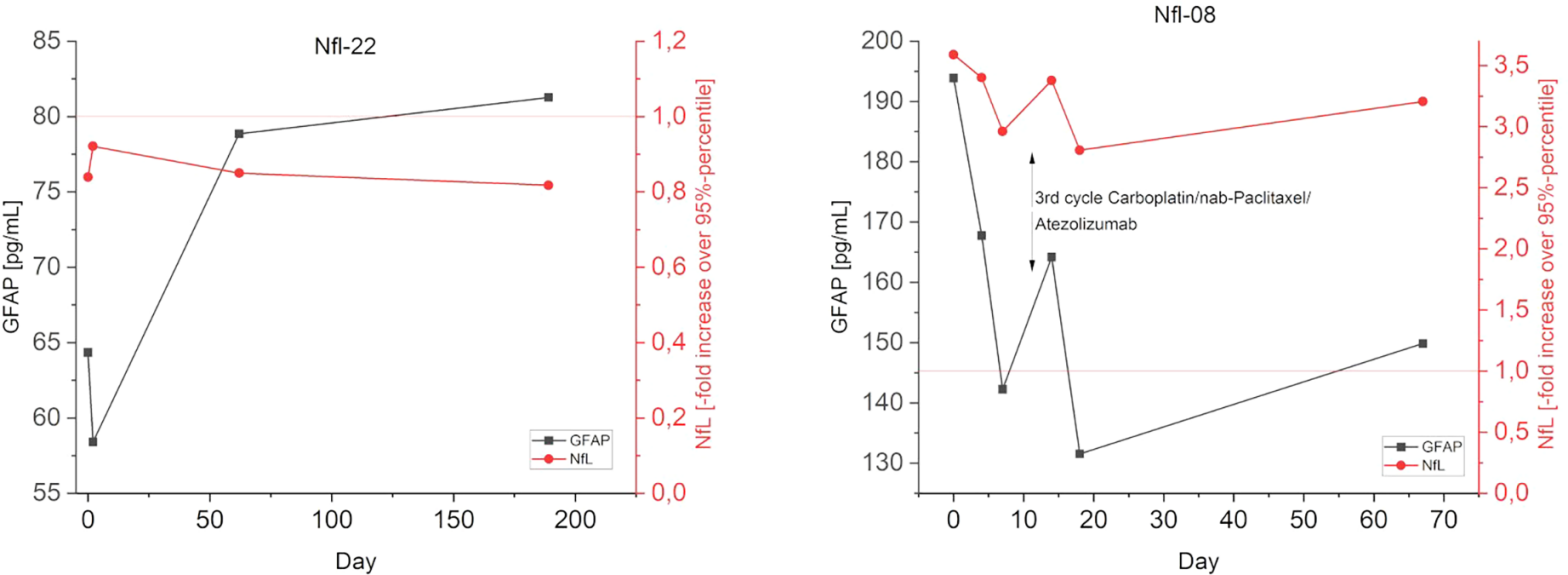
Patients NfL-22 (left) and NfL-08 (right).

However, the favourable NfL response after SRT and its correlation with unremarkable MRI follow-up in these two patients were not observed across the entire cohort. For example, NfL-22, a 65-year-old patient receiving SRT to four cerebral metastases from NSCLC on three consecutive days, showed NfL values below the 95th percentile of the reference distribution, with no clear trend after therapy. NfL-08, a 67-year-old patient with NSCLC treated with SRS for one cerebral metastasis, exhibited relatively stable NfL levels despite good response on follow-up MRI. A small peak in both NfL and GFAP was observed on day 14, coinciding with administration of the third chemotherapy cycle (carboplatin/nab-paclitaxel/atezolizumab) three days earlier (**Figure 5**).

**Figure 6:**
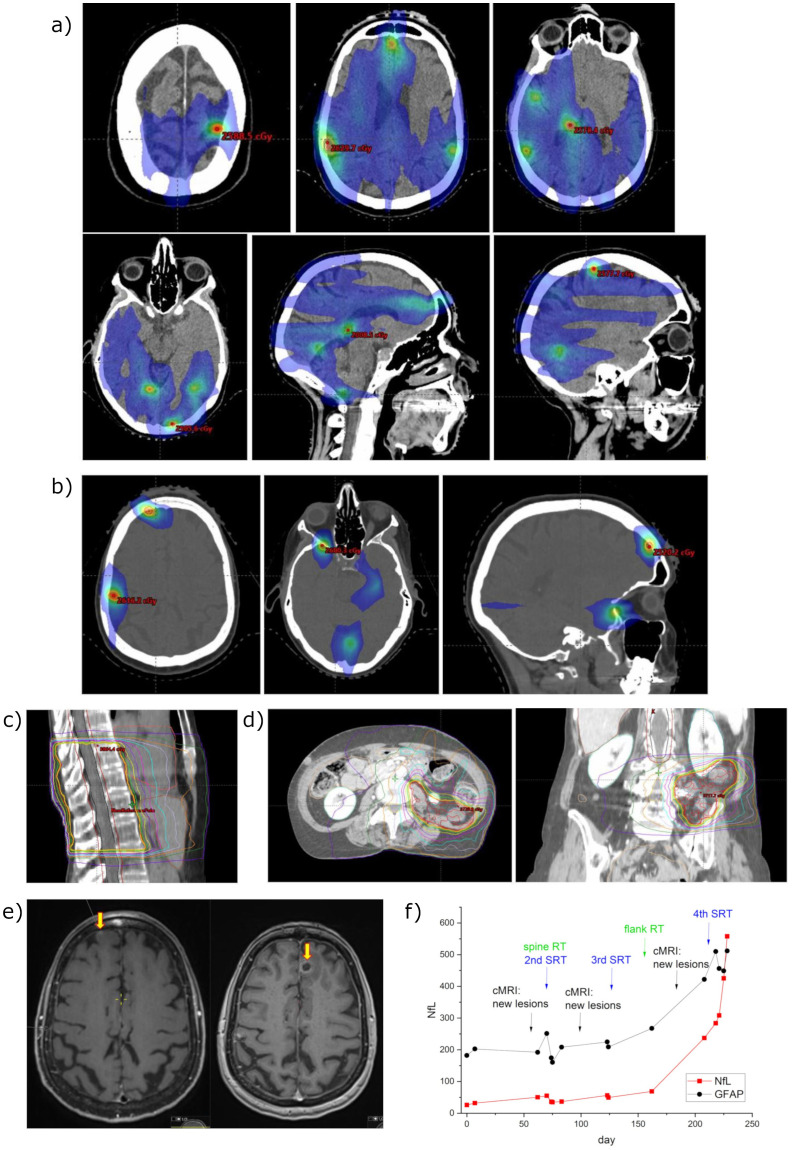
Patient NfL-13, presenting with 14 cerebral metastases from malignant melanoma. **(a)** Treatment plan for first course of stereotactic radiotherapy (14 metastases treated with a single isocenter in a single session to receive 20 Gy to the encompassing 80 % isodose). **(b)** Treatment plan for second course of stereotactic radiotherapy to 8 new lesions (detected on day 54, treated on day 70), again with single-session radiosurgery with 20 Gy. **(c)** Treatment plan for a bone metastasis at the thoracolumbar junction, treated from day 68 to 83 with 12 fractions of 3 Gy (VMAT). **(d)** Treatment plan for a soft-tissue metastasis at the left flank, treated with 12 fractions of 3 Gy from days 123 to 162. **(e)** Example cMRT slice on day 54 (left) and 99 (right). The image shows that a lesion visible on day 54, which received stereotactic radiosurgery on day 70, responded well and regressed, while a new lesion appeared on day 99. The patient therefore received a third course of stereotactic radiotherapy, which is not shown in the figure. **(f)** NfL and GFAP values for this patient over the course of the treatment courses and follow-up.

NfL-13 (**Figure 6**): This was a 65-year-old patient with malignant melanoma, presenting with 14 cerebral metastases without neurological signs or symptoms. The treatment plan is shown in **Figure 3**, using Brainlab Elements MultipleBrainMets with 6 MV FFF photons delivered via dynamic conformal arc therapy (DCA) with a single isocentre and a prescribed dose of 25 Gy (20 Gy to the encompassing 80% isodose). The patient tolerated the treatment well, and follow-up cMRI on day 54 demonstrated good response of all irradiated metastases. However, eight new lesions had appeared. This was accompanied by an increase in NfL from day 7 to day 62. Therefore, a second course of SRT was administered on day 70, again using single-isocentre DCA with 6 MV FFF photons and a single fraction of 20 Gy prescribed to the 80% isodose surrounding the lesions. In parallel, an osseous lesion at the thoracolumbar junction was treated palliatively from day 68 to day 83 with 36 Gy delivered in 12 fractions using volumetric modulated arc therapy (VMAT), without specific dose sparing of the spinal canal (dose distributions are shown in **Figures 6B, C**). The subsequent cMRI follow-up on day 99 again demonstrated good response of most treated lesions, but also revealed seven new metastases (example slice shown in **Figure 6D**). At this time point, a slight increase in NfL was observed. A third course of SRT was therefore delivered on day 123, identical in treatment technique and dose prescription to the previous courses. The cMRI follow-up on day 144 demonstrated stable disease without new lesions. A modest increase in NfL from day 123 to day 162 was noted; however, as this remained below 20%, it may not be clinically meaningful. In addition, a soft-tissue metastasis at the patient’s left flank was treated palliatively with 36 Gy in 12 fractions from day 153 to day 171 (**Figure 6E**), which may have confounded the NfL value on day 162 due to possible peripheral neuropathic effects. The cMRI on day 184 demonstrated a new metastasis, and a planning cMRI was therefore scheduled for a fourth SRT course. This imaging revealed 28 new lesions, corresponding to a marked rise in NfL on day 208. Despite a further SRT attempt to control these metastases using several fractionation schemes, the patient died approximately six weeks after the last treatment due to non-neurological causes.

For this patient, NfL and GFAP showed a close temporal correlation during each increase and decrease (**Figure 6F**). Each occurrence of new metastases was associated with an increase in NfL and GFAP, whereas each course of SRT was accompanied by a decrease. In the final cMRI, when extensive intracranial progression was observed, NfL and GFAP increased markedly, with NfL even exceeding GFAP levels, which were generally higher than those of NfL.

**Figure 7:**
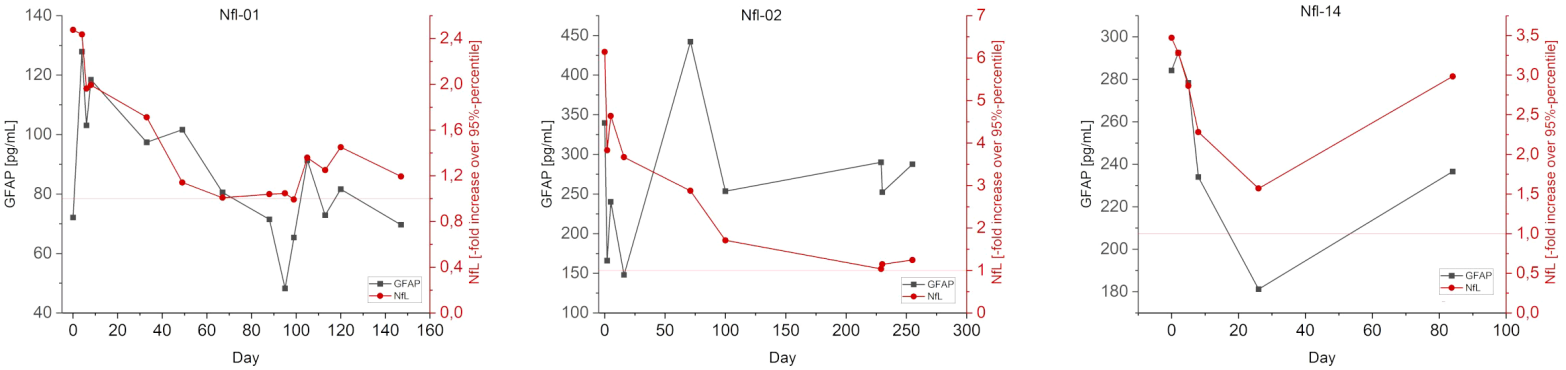
NfL and GFAP levels in patients NfL-01, NfL-02, and NfL-14; all patients received stereotactic radiotherapy after resection of at least one cerebral metastasis.

In patients undergoing stereotactic treatment of brain metastases after resection, all baseline values were elevated as a consequence of the surgical procedure. Overall, a consistent pattern was observed, with NfL values decreasing after SRT and reaching or nearly reaching the 95th percentile during long-term follow-up (NfL-1, NfL-2, NfL-14; **Figure 7**).

In NfL-1 and NfL-14, NfL serum levels decreased after treatment. In NfL-1, NfL remained close to the 95th percentile, while MRI follow-up demonstrated good response without intracranial progression or relapse. NfL-14 showed decreasing NfL values until an increase occurred on day 84. In this patient, an adenocarcinoma of the gastro-oesophageal junction had been diagnosed as a second malignancy, and perioperative chemotherapy according to the FLOT protocol (5-fluorouracil, leucovorin, oxaliplatin, and a taxane) had been initiated by that time, which may explain the increase in NfL despite stable cMRI findings.

For NfL-02, NfL decreased to approximately the 95th percentile on day 229 after treatment (one lesion treated with surgery followed by post-operative SRT and one lesion treated with SRT alone). However, on day 215, four new lesions were detected on MRI (no NfL measurement was obtained on that day), and these were treated with SRT (MultipleBrainMets) on day 229. Notably, no increase in NfL serum level was observed on day 229 relative to the 95th percentile; therefore, these new lesions would not have been identified by NfL measurements. The NfL level subsequently remained close to the 95th percentile, and good intracranial response was observed during follow-up.

**Figure 8:**
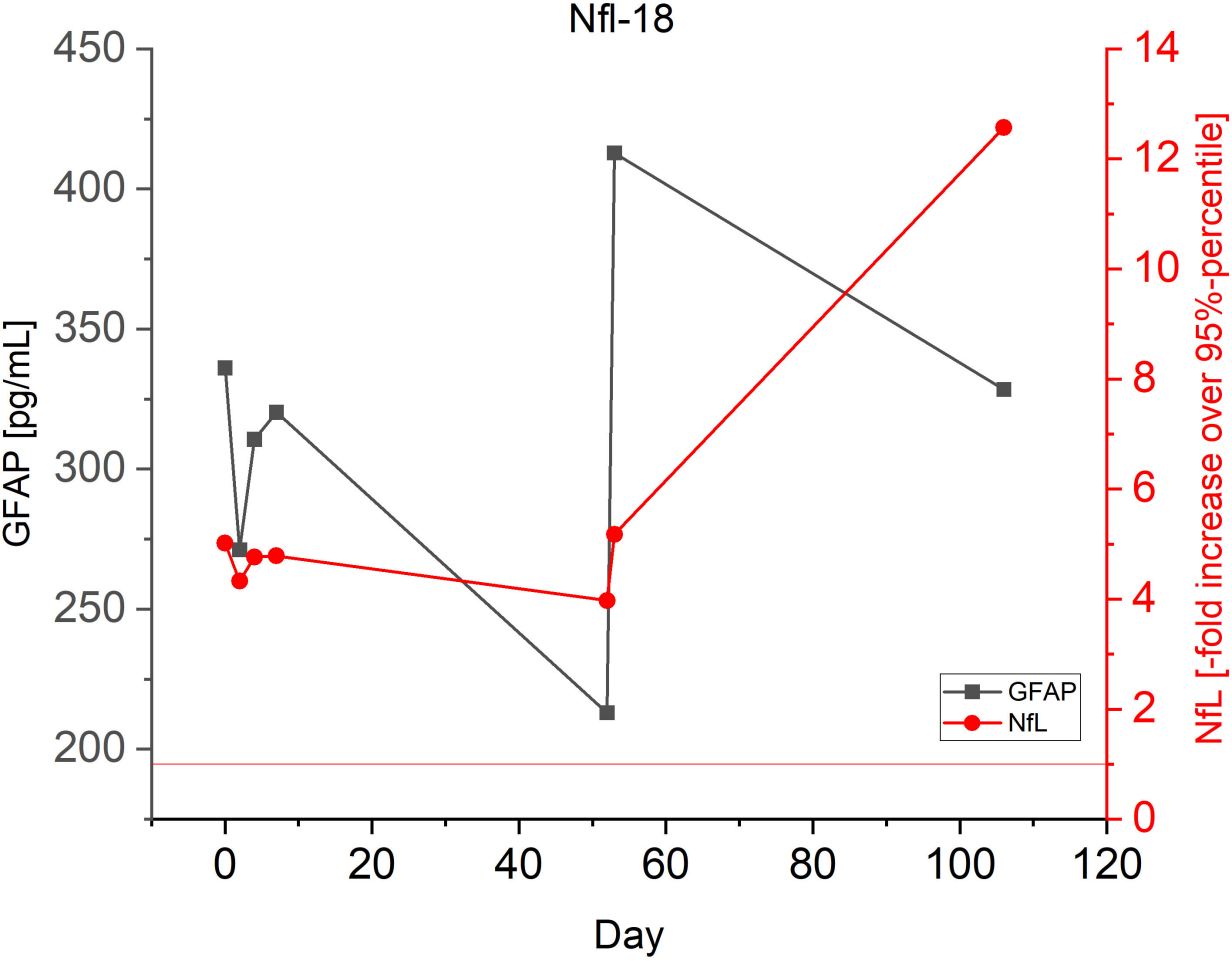
NfL and GFAP levels in patient NfL-18 (post-operative radiotherapy on day 0 and radiotherapy without prior resection on day 53).

The only patient in whom NfL remained significantly elevated after surgery and post-operative SRT was NfL-18 (**Figure 8**). In this patient, in addition to the treated metastasis, a second intracerebral lesion was known but did not receive upfront treatment, as the tumour board recommended surveillance. On the subsequent MRI, slight progression of this lesion was observed, and SRT was therefore performed for the second metastasis. Unfortunately, follow-up examinations were not performed at close intervals, and it cannot be determined whether NfL decreased after the second SRT on day 53. The next measurement on day 106 showed a marked increase, with NfL values even exceeding GFAP levels. Six days earlier, cMRI follow-up had still demonstrated good intracranial disease control; however, eight weeks later, multiple new metastases were detected on MRI, and the patient died shortly thereafter. It is conceivable that the elevated NfL value may have reflected widespread intracerebral dissemination before it became radiologically apparent on cMRI.

**Figure 9:**
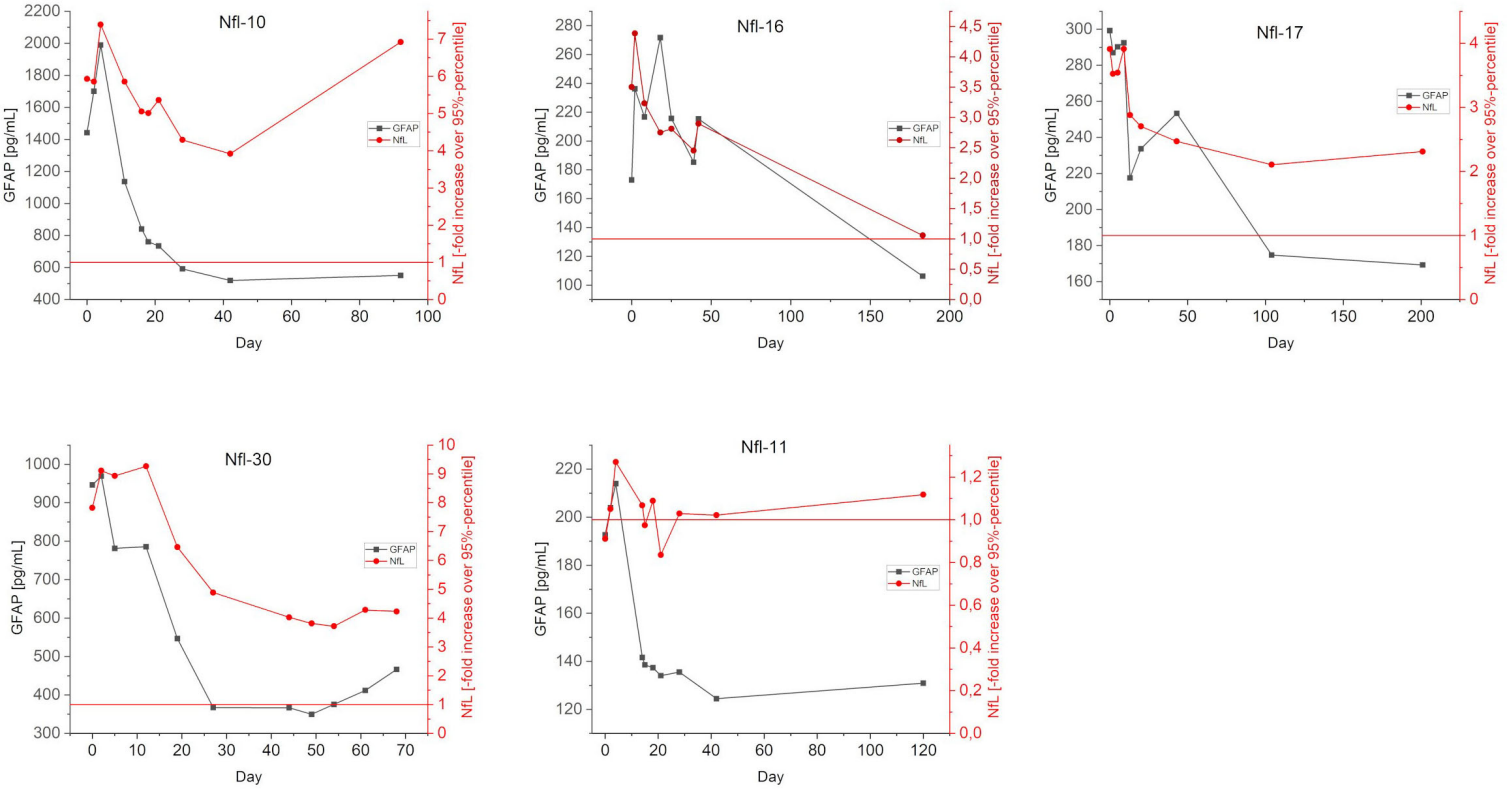
NfL and GFAP levels in patients NfL-10, NfL-16, NfL-17, and NfL-30 (GBM), and NfL-11 (DGONC).

For patients with glioblastoma (**Figure 9**), NfL was elevated at the start of treatment and decreased thereafter. The only exception to this pattern was NfL-11, the patient with DGONC, whose values remained close to the 95th percentile of NfL prior to, during, and after treatment, without a discernible trend. MRI follow-up demonstrated good response, and the patient’s clinical condition remained stable. For all other patients, the decrease in NfL correlated with favourable response on MRI follow-up examinations, although NfL did not decline to the 95th percentile in all cases. Follow-up duration was insufficient to detect MRI-confirmed relapse in these patients; therefore, it remains unclear whether such relapse would have been accompanied by an increase in NfL. However, the only patient with a renewed rise in NfL (NfL-10, day 92) showed residual disease on MRI performed on the same day. Clinically, this patient presented with impaired performance status following a focal seizure and rapid progression of neurological symptoms. In the subsequent weeks, the patient developed hemineglect, became bedridden, and died under best supportive care.

**Figure 10:**
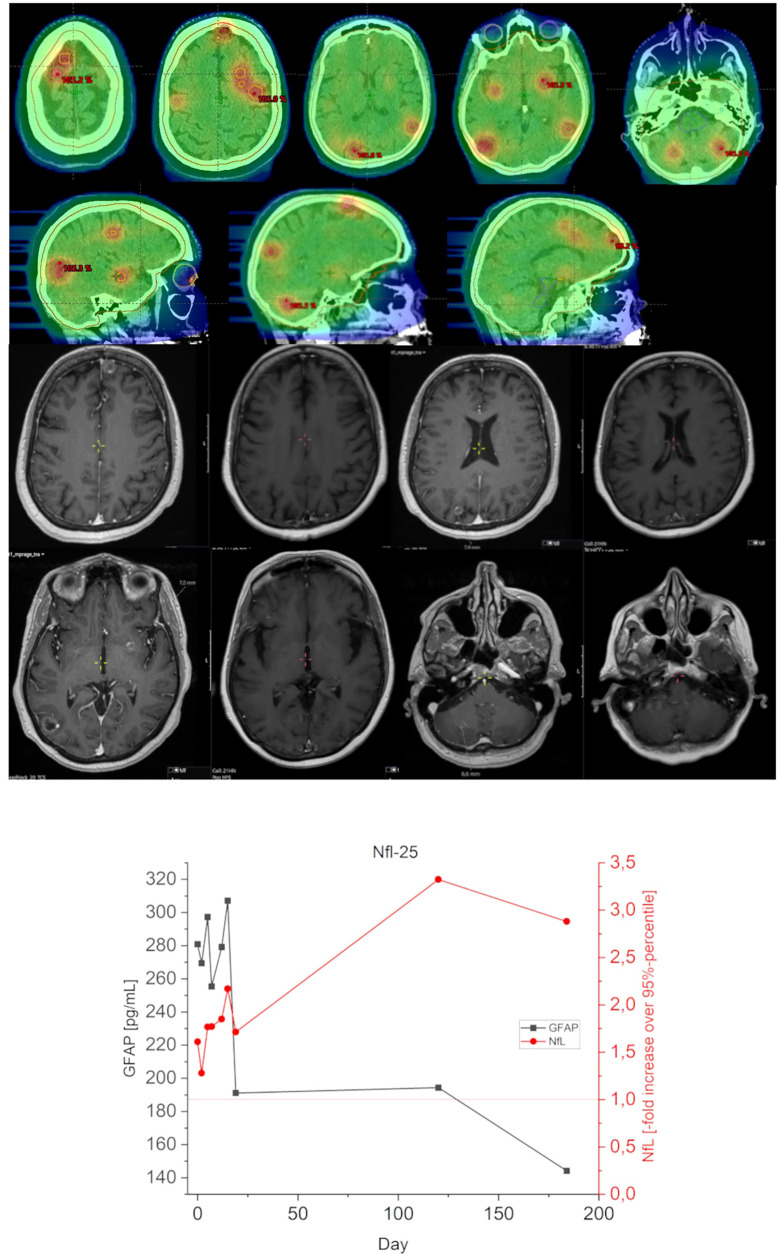
Patient NfL-25 treated with WBRT plus SiB to 15 brain metastases, with cranial MRI at first follow-up showing almost complete disappearance of all macroscopic lesions. NfL and GFAP serum values are displayed at the bottom of the figure.

Finally, patients treated with whole-brain irradiation are considered. NfL-25 was a 58-year-old female patient with extensive-stage SCLC, who received first-line chemotherapy followed by consolidative thoracic radiotherapy in June 2023 and subsequently therapeutic WBRT with a simultaneous integrated boost to 15 macroscopic cerebral metastases (volumetric modulated arc therapy with 6 MV photons; 12 × 3 Gy to the whole brain and 12 × 4.25 Gy to the metastases). The treatment plan is shown in **Figure 10**, together with the corresponding NfL and GFAP serum values. While GFAP showed some variation, with an overall decreasing trend, NfL remained relatively constantly elevated until a pronounced increase was observed on day 120. MRI after completion of radiotherapy demonstrated a very good response, with almost complete regression of all visible cerebral metastases (**Figure 10** compares the planning MRI from day −6 with the first follow-up on day 82). Unfortunately, no serum sample was obtained on that day. The next available sample was from day 120 and showed an increase in NfL. Available clinical data from three months after this measurement indicated that the patient had developed multiple new intracranial metastases and was scheduled to present to our department for evaluation of cranial re-irradiation. After this entry, the patient was lost to follow-up. It is conceivable that the rise in NfL at day 120 may already have reflected the emergence of new intracranial lesions; however, this cannot be confirmed due to the absence of contemporaneous imaging.

**Figure 11:**
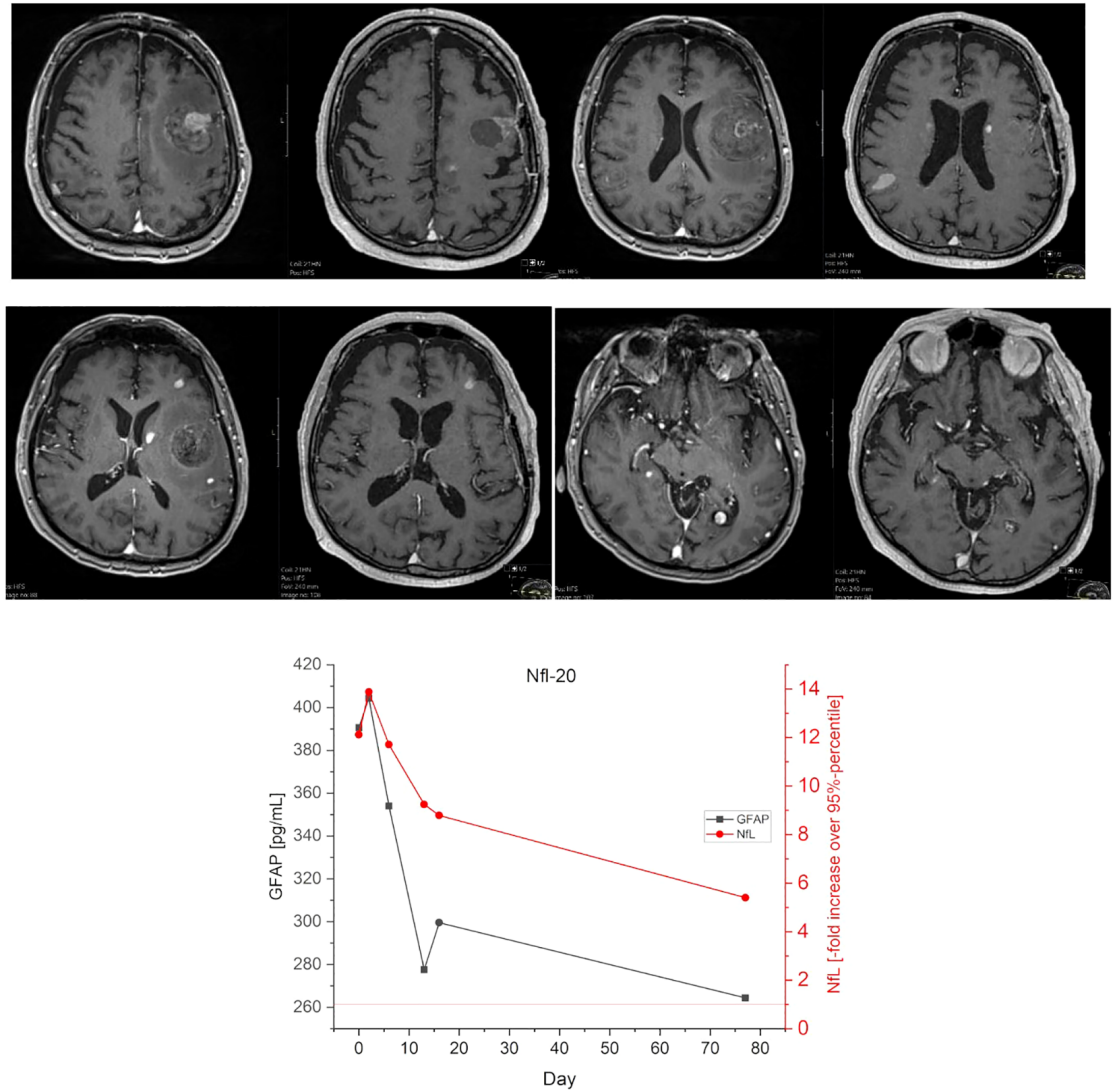
Patient NfL-20, who underwent surgical resection of one brain metastasis followed by WBRT, demonstrating very good response of most lesions on follow-up imaging.

In contrast to this patient, NfL-20 was a 77-year-old male patient with 16 brain metastases from malignant melanoma, who underwent surgical resection of the largest (symptomatic) metastasis followed by therapeutic cranial irradiation (12 × 3 Gy). In this patient, the baseline NfL value was considerably higher than in NfL-25, which is plausible in light of the preceding surgical procedure. After treatment, both NfL and GFAP showed a consistent decrease, corresponding to good response (although not complete disappearance of the lesions) on follow-up MRI on day 42 (last available MRI) (**Figure 11**).

Sufficient follow-up data were available for only one patient receiving PCI, namely NfL-27. In this patient, follow-up MRI remained unremarkable throughout the period during which blood samples were obtained. A small peak in NfL and GFAP was observed on day 100, which subsequently declined and did not correlate with the appearance of new metastases on MRI. As this patient received maintenance immunotherapy outside our clinic, the cause of this temporary increase in serum parameters cannot be attributed to a specific factor.

## 4 Discussion

This investigation was initiated with three research questions, which are addressed separately below in light of the available literature.

### 4.1 Are baseline levels of these markers elevated before the start of radiotherapy?

NfL serum levels were elevated above the age-adjusted median reference value in all patients in this cohort, thereby confirming previous findings. Hepner et al. (41) and Winther-Larsen et al. (42) were the first to describe significantly increased NfL and GFAP levels in patients with primary or secondary brain malignancies. Kim et al. (50) reported age-specific cut-off values for NfL and GFAP. When comparing these thresholds with baseline samples from patients who had not undergone surgery, all patients with metastases (treated with WBRT or SRT thereafter), except for NfL-39 (NfL 17.2 pg/mL, GFAP 62.2 pg/mL despite approximately 23 supra- and infratentorial metastases) and NfL-22 (NfL 16.8 pg/mL, GFAP 64.4 pg/mL with two cerebral metastases), exceeded the NfL cut-off value. NfL-29, who received PCI without known cerebral metastases, remained below these thresholds. However, NfL-23 (also receiving PCI without known metastases) exceeded the cut-off proposed by Kim et al. (50). Among these patients, those with multiple metastases requiring WBRT also exceeded the GFAP threshold reported by Kim et al. (50), whereas patients receiving SRT for a limited number of metastases exhibited lower GFAP values. It appears plausible that patients receiving PCI without known cerebral metastases had the lowest baseline NfL and GFAP values; however, it is noteworthy that even these levels were elevated above the reference range for NfL (and, for GFAP, of a similar magnitude to those observed in patients with known metastases treated with SRT). This finding may be explained by prior chemotherapy, or it may reflect the presence of micrometastases that were not yet detectable on MRI.

In conclusion, although sensitivity remained below 100%, 17 of 19 patients with known metastases in this cohort would have been identified on the basis of NfL and GFAP values using the cut-off proposed by Kim et al. (50). Although it is conceivable that the volume of intracranial disease may influence the magnitude of NfL and GFAP elevation, the present cohort is too small to conclusively test this hypothesis.

### 4.2 How do NfL and GFAP levels change over the course of radiotherapy? Can an increase in serum levels be observed during or after radiotherapy, and, if so, does this differ between treatment regimens (e.g., WBRT vs. SRT)?

When comparing NfL and GFAP serum levels obtained a few days after initiation of radiotherapy with baseline values, no significant increase was observed. In patients who had undergone previous surgery, a decrease was noted.

The declining trend in NfL and GFAP measurements following surgery is expected and is consistent with observations in patients with acute head injury.

It has been reported that serum NfL and GFAP exhibit distinct kinetic profiles following acute neuronal injury, with NfL levels elevated on day one after trauma and continuing to rise for two to three weeks, remaining increased for months, whereas GFAP concentrations typically peak within 20–24 h and gradually decline over approximately 72 h, with an apparent plasma half-life of 24–48 h (31). Therefore, any elevations in NfL and GFAP as a potential consequence of neuronal damage caused by cerebral irradiation would be expected to become detectable in serum one to two days after radiation exposure, but to decline according to different temporal patterns.

In patients receiving radiotherapy alone, an increase in NfL and GFAP within one to two days had been anticipated. Contrary to this hypothesis, no significant increase in NfL or GFAP could be attributed to radiotherapy. This finding applied across all treatment regimens: stereotactic treatment, PCI or WBRT (with or without hippocampal sparing and with or without simultaneous integrated boost), as well as VMAT for glioblastoma.

To our knowledge, only one previous study has examined NfL and GFAP behaviour during radiotherapy, albeit with a considerably coarser temporal resolution of measurements. Abu-Rumeileh et al. (51) published data from 33 patients with malignant gliomas undergoing surgical and radiation treatment, demonstrating pre-therapeutic elevations of NfL and GFAP and a tendency towards decreasing GFAP levels after radiotherapy, while no significant change in NfL levels was observed. These authors reported data at only three time points (t1: before surgery; t2: after surgery and before initiation of radiotherapy; t3: after radiotherapy), so that short-term effects following individual radiotherapy fractions could not be assessed.

In the present study, changes in NfL and GFAP serum levels were analysed in patients treated with a variety of radiotherapy protocols for different malignant diseases of the central nervous system (CNS). Comparisons of NfL and GFAP levels within the different treatment groups using the Wilcoxon test showed no significant changes from baseline to early treatment (one to four days after initiation of radiotherapy). Nor was a delayed increase observed from baseline to approximately 11 days (range 6–14 days) after initiation of treatment in any group.

When examining individual patient cases, certain patterns may be tentatively suggested. Patient NfL-01 received a single session of radiosurgery delivering 25 Gy (Vol_PTV_ 0.1 cm^3^) 25 days (designated d0) after surgery for a frontal NSCLC metastasis, as well as fSRT delivering 3 × 9 Gy (Vol_PTV_ 4.1 cm^3^) to another frontal metastasis (d5–7). In this patient, NfL was elevated approximately 2.5-fold above the reference value at the initiation of radiotherapy (d0) and decreased thereafter, whereas GFAP increased 1.77-fold from d0 to d4. Based on the previously described kinetic profiles of NfL and GFAP, this observation may be interpreted as follows: the elevated NfL level at baseline may have reflected the preceding surgical intervention 25 days earlier, whereas GFAP, if initially elevated, may already have declined. Consequently, the subsequent increase in GFAP could represent a transient response to radiotherapy. In another case (NfL-24), a patient received radiosurgery at a dose of 25 Gy to two lesions with PTVs of 4.3 and 1.1 cm^3^, respectively, without prior surgery. In this instance, a 1.77-fold increase in GFAP was observed by day 5 after irradiation, followed by an apparent biphasic decline, while NfL increased only 1.09-fold, which falls within the 20% methodological variability of the assay. However, in a further patient (NfL-26) treated with single-session radiosurgery delivering 25 Gy to a smaller PTV of 0.1 cm^3^, no increase in either GFAP or NfL was observed.

Similarly, in some patients treated with fractionated radiotherapy for GBM (VMAT to 60 Gy in 30 fractions) and in patient NfL-20 receiving WBRT after surgery, a transient increase in both NfL and GFAP was observed during the first days of radiotherapy, followed by a more gradual decline. Therefore, it is conceivable that, in certain individuals, a modest and short-lived elevation in these serum markers may occur shortly after initiation of radiotherapy, followed by a marked decrease correlating with therapeutic response. However, analysis of the data from the entire cohort did not demonstrate statistical significance, suggesting that these findings may represent individual variability rather than a consistent treatment effect.

Since increases in serum concentrations have been reported to correlate with the extent of cerebral injury in other disease contexts, the present findings suggest that any acute neuronal or glial injury induced by radiotherapy is likely to be limited.

### 4.3 What is the behaviour of these values during longer follow-up? Do they return to normal? Can they be used as early markers of relapse or disease progression?

During long-term follow-up, decreasing NfL and GFAP levels were generally associated with therapeutic response. The most pronounced increases in serum NfL levels observed (e.g. NfL-13) were followed by the detection of multiple new cerebral metastases. These findings suggest that NfL and GFAP may represent suitable candidates for blood-based monitoring and potentially for early detection of new lesions, which is consistent with the observation that cerebral lesions are associated with elevated serum markers (see question 1). However, as not all recurrences were reflected by corresponding increases in these biomarkers, the relationship between intracranial tumour burden and serum marker concentrations, and thus the overall sensitivity of these markers, remains unclear and warrants further investigation.

In the case of NfL-18 (post-surgical fSRT for a cerebral NSCLC metastasis), a steep rise in NfL levels was noted six days after MRI had demonstrated good disease control, but this was followed by detection of a large number of new metastases on MRI eight weeks later. This observation may suggest that increases in NfL levels precede radiological detectability of lesions on MRI. A comparable “lead time” between NfL elevation and MRI diagnosis of brain metastases of one to five months (median three months) was reported by Winther-Larsen et al. (42), which is consistent with the present findings. While this raises the possibility of adapting MRI scheduling according to biomarker increases during follow-up, it also prompts consideration of the optimal interval between detection of serum marker changes and subsequent MRI examinations in order to minimise false-negative imaging findings.

Despite the anecdotal nature of these observations, two instances were noted in which NfL serum levels exceeded those of GFAP (NfL-18, day 106, and NfL-13, day 228), and both patients died shortly thereafter. This finding raises the possibility that the magnitude of serum marker elevation may also carry prognostic information, as previously suggested by Winther-Larsen and Gottiparthy et al. (42, 52).

### 4.4 Strengths and limitations of this study

The main limitation of this study is the small sample size, which restricts statistical power, as well as the heterogeneity of the cohort and the variability in follow-up duration. To some extent, heterogeneity of the study cohort was intentional in order to enable comparison between patients undergoing radiosurgery, whole-brain irradiation, and treatment for glioblastoma. However, during the recruitment period, our department introduced single-isocentre stereotactic radiotherapy for multiple brain metastases. On the one hand, this resulted in increased heterogeneity among patients with brain metastases, including a higher number of lesions and different fractionation regimes. On the other hand, it led to a marked reduction in the number of patients receiving whole-brain irradiation. In order to accrue a sufficient number of patients within the study period, all patients undergoing whole-brain radiotherapy were included, irrespective of whether PCI or therapeutic WBRT was administered, and regardless of the use of SiB concepts or hippocampal sparing. Furthermore, funding was limited to laboratory assays, and patient travel and insurance costs were not covered. Consequently, blood samples could only be obtained when patients attended the clinic for routine follow-up examinations or for systemic therapy administered by other departments. As a result, several planned sampling time points were not available for analysis.

In summary, this study is limited by a relatively small and heterogeneous cohort of patients. Only a few comparisons reached statistical significance, as detailed above. The heterogeneity may also restrict external validity, and the findings may not be fully generalisable to other clinical settings. Nevertheless, NfL and GFAP levels were elevated prior to radiotherapy, which is consistent with previous investigations. Furthermore, as a novel observation, the present results suggest that no measurable increase in NfL or GFAP occurs during radiotherapy, irrespective of the treatment regime applied.

Despite these limitations, the dataset represents routine clinical practice at our department and comprises a representative cohort of unselected patients receiving cerebral radiotherapy. To our knowledge, it constitutes the most comprehensive and most densely sampled cohort to date with respect to NfL and GFAP measurements in patients undergoing cerebral radiotherapy. The findings confirm elevated NfL and GFAP serum levels in patients with primary or secondary CNS tumours, their decline in association with treatment (surgery and radiotherapy, combined with chemotherapy for GBM), and their dynamic behaviour during follow-up and potential relapse.

There remains a lack of widely accepted reference values for GFAP. Although recent publications (31) have discussed the feasibility of using areas under the curve of GFAP levels to predict outcomes in patients with traumatic brain injury without prior baseline measurements, these studies also emphasise the need for age-adjusted cut-offs and consideration of inter-platform variability. With regard to NfL levels, age-related reference values published by Simrén et al. (49) were used as baseline comparators; however, these values were established using plasma rather than serum samples, and other factors known to influence NfL concentrations, such as body mass index, were not accounted for. Nevertheless, in the context of employing serum biomarkers for detection of relapse after oncological treatment, relative longitudinal changes may be more informative than direct comparison with reference values derived from healthy individuals. To support the robustness of the analytical approach, it should be noted that the Simoa (single-molecule array) HD-X platform is currently regarded as the gold standard for ultra-sensitive protein detection. The principal strength of this assay lies in its ability to reliably quantify NfL and GFAP in peripheral blood (serum or plasma), where concentrations are typically approximately 100-fold lower than in cerebrospinal fluid and below the detection limits of conventional enzyme-linked immunosorbent assays. This transition towards non-invasive biomarker monitoring represents a well-established and technically robust advancement in neurodiagnostics.

Since NfL is expressed in neurons of both the peripheral and central nervous systems, and previous studies have shown that axonal damage following chemotherapy leads to increases in serum NfL levels (53, 54), a further limitation of the present study—and of NfL as a biomarker of neuronal injury in general—is the potential confounding effect of systemic therapy. Kim et al. (54) reported increases in NfL during chemotherapy that were not paralleled by GFAP elevations. Moreover, NfL concentrations may be increased in various neurological conditions, such as peripheral neuropathy, which may coexist in patients with cancer due to comorbidities including diabetes mellitus or alcohol misuse. If future studies confirm that GFAP is not elevated under such circumstances, combined assessment of both markers may help to reduce diagnostic ambiguity [see, for example (55)].

### 4.5 Significance of results for radiotherapy clinical patient management

The management of patients with brain metastases is currently undergoing a paradigm shift, as stereotactic radiotherapy is increasingly preferred over whole-brain irradiation, even in the presence of multiple metastases or primary tumours with a high propensity for cerebral dissemination, such as small-cell lung cancer (56–58). While this strategy achieves excellent local control of treated lesions, it is associated with an increased risk of distant intracranial relapse, necessitating repeated interventions. Non-inferior overall survival can only be maintained in this context if frequent MRI examinations are performed, often at intervals of eight weeks or less.

In routine clinical practice, this approach may be challenging, firstly because obtaining MRI appointments at such short intervals may not be feasible, and secondly because repeated imaging can impose considerable psychological and physical burden on patients. Consequently, even in highly developed countries, some centres continue to favour whole-brain irradiation for patients with four or more brain metastases, based on the rationale that this may provide comprehensive intracranial treatment and reduce the need for intensive follow-up. Repeated blood sampling represents a less burdensome alternative and may allow postponement of MRI examinations in patients with stable and low serum marker levels, while identifying those more likely to benefit from early re-treatment. Therefore, if validated in larger prospective studies, incorporation of these biomarkers into follow-up strategies could represent a practice-changing approach, potentially reducing healthcare costs and facilitating broader implementation of stereotactic treatment for a larger cohort of patients.

## 5 Conclusions and interpretation

Contrary to expectations, neither NfL nor GFAP was significantly influenced by radiotherapy, irrespective of whether whole-brain irradiation, stereotactic treatment, or partial-brain high-dose irradiation for glioblastoma therapy was applied. Even ablative doses delivered during radiosurgery did not provoke an increase in NfL or GFAP levels. Given that NfL has been demonstrated to be highly sensitive to neuronal injury in various clinical contexts, these findings suggest that even stereotactic radiosurgery doses may be less detrimental to neuronal integrity than might be assumed based on their therapeutic efficacy.

These results are presented in the conviction that negative findings warrant publication alongside positive results. In a second step, an exploratory analysis of individual patient cases was conducted. In line with previous studies, NfL and GFAP levels were confirmed to be elevated above reference values prior to therapy, and increases in these biomarkers were associated with recurrence of cerebral lesions. Although such associations may have been anticipated, the present findings replicate these observations in a cohort of patients all of whom underwent cerebral radiotherapy. Furthermore, patients exhibiting a pronounced relative increase in NfL compared with GFAP at certain time points appeared to experience rapid clinical deterioration, an observation not previously reported.

Our conclusions are therefore twofold. First, cerebral radiotherapy does not appear to compromise neuronal and glial integrity to an extent sufficient to induce measurable increases in NfL or GFAP levels. Second, further investigation is warranted to determine whether NfL- and GFAP-based follow-up of patients treated for cerebral metastases, with prioritised MRI appointments in cases of rising serum markers, could represent a clinically feasible alternative to the current standard, potentially reducing resource utilisation while maintaining oncological safety in surveillance strategies.

## Data Availability

The original contributions presented in the study are included in the article/supplementary material. Further inquiries can be directed to the corresponding authors.
